# Genome editing for sickle cell disease: still time to correct?

**DOI:** 10.3389/fped.2023.1249275

**Published:** 2023-11-02

**Authors:** Giulia Ceglie, Marco Lecis, Gabriele Canciani, Mattia Algeri, Giacomo Frati

**Affiliations:** ^1^ Cell and Gene Therapy for Hematological Disorders Unit, Department of Oncology-Hematology, Ospedale Pediatrico Bambino Gesù, Rome, Italy; ^2^ Department of Systems Medicine, University of Rome Tor Vergata, Rome, Italy; ^3^ Pediatric Unit, Modena University Hospital, Modena, Italy; ^4^ Residency School of Pediatrics, University of Rome Tor Vergata, Rome, Italy

**Keywords:** sickle cell disease, gene editing, fetal hemoglobin reactivation, globin genes regulation, gene therapy, CRISPR/Cas9

## Abstract

Sickle cell disease (SCD) is an inherited blood disorder, due to a single point mutation in the β-globin gene (*HBB*) leading to multisystemic manifestations and it affects millions of people worldwide. The monogenic nature of the disease and the availability of autologous hematopoietic stem cells (HSCs) make this disorder an ideal candidate for gene modification strategies. Notably, significant advances in the field of gene therapy and genome editing that took place in the last decade enabled the possibility to develop several strategies for the treatment of SCD. These curative approaches were firstly based on the correction of disease-causing mutations holding the promise for a specific, effective and safe option for patients. Specifically, gene-editing approaches exploiting the homology directed repair pathway were investigated, but soon their limited efficacy in quiescent HSC has curbed their wider development. On the other hand, a number of studies on globin gene regulation, led to the development of several genome editing strategies based on the reactivation of the fetal γ-globin gene (*HBG*) by nuclease-mediated targeting of *HBG*-repressor elements. Although the efficiency of these strategies seems to be confirmed in preclinical and clinical studies, very little is known about the long-term consequences of these modifications. Moreover, the potential genotoxicity of these nuclease-based strategies must be taken into account, especially when associated with high targeting rates. The recent introduction of nuclease-free genome editing technologies brought along the potential for safer strategies for SCD gene correction, which may also harbor significant advantages over *HBG*-reactivating ones. In this Review, we discuss the recent advances in genome editing strategies for the correction of SCD-causing mutations trying to recapitulate the promising strategies currently available and their relative strengths and weaknesses.

## 1. Introduction

**Figure 1:**
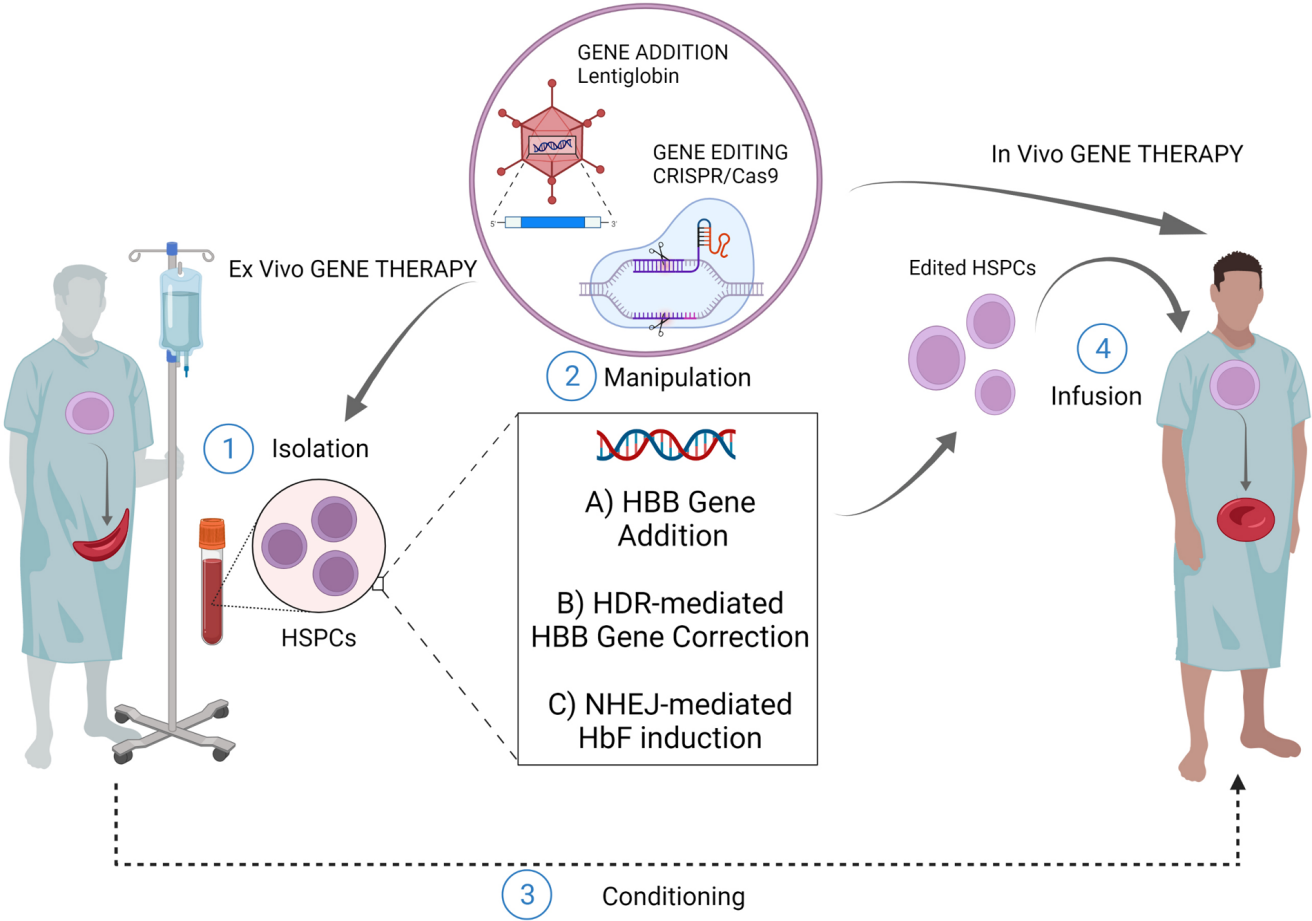
Gene therapy/editing for SCD. The *ex vivo* gene therapy relies upon isolation of patients HSPCs, their manipulation and subsequent infusion of the cellular product after a conditioning regimen. The most exploited strategies for this scope are: *ex vivo* gene addition based on virus-mediated delivery of a functional copy of *HBB* gene (e.g. lentiglobin); *ex vivo* genome editing (nuclease based HDR-mediated correction of the *HBB* gene); *ex vivo* gene editing (nuclease based NHEJ-mediated HbF induction) The *in vivo* gene therapy relies upon direct infusion of the editing machinery to target patient resident HSPCs, without isolation-manipulation-conditioning and re-infusion. (Created with BioRender.com).

Sickle cell disease (SCD) is a disorder characterized by the inheritance of a single base substitution in the first exon of the β-globin gene (*HBB*). This single point mutation causes an aminoacidic replacement of the hydrophilic glutamic acid with a hydrophobic valine at the 7th codon of β-globin chain. Whether inherited in a homozygous manner or with another mutation in the *HBB*, this substitution significantly alters the function of hemoglobin constituting a pathological form of hemoglobin, defined as sickle hemoglobin (*α*_2_βs_2_, HbS). The mutant HbS undergoes polymerization and aggregation upon deoxygenation, thus conferring the typical sickle shape to the Red Blood Cells (RBCs). This distortion results in decreased RBCs' survival and several downstream clinical manifestations, including chronic anemia, pain events, stroke, multiorgan damage and failure, and premature mortality (1). The two cornerstones of SCD management, blood transfusions and Hydroxyurea, have greatly increased the survival and quality of life of patients but do not fully eliminate the consequences of the disease (2). So far, the only curative treatment is represented by allogeneic hematopoietic stem cell (HSC) transplantation, when a compatible donor is available. Nevertheless, less than 15% of patients with SCD have an appropriately matched donor (3). In this context, gene therapy based on the autologous transplantation of genetically modified HSC constitutes a promising strategy to overcome the major barriers in the cure of SCD (Figure 1). Classical viral-mediated gene addition protocols, based on the delivery of a functional copy of the *HBB* gene, have been successfully translated into several clinical trials mostly resulting in a decreased transfusion need for patients (4–7). However, some limitations (e.g., limited transduction efficiency and engraftment capability of HSC; variable Hb production levels; high manufacturing costs of the viral vector) still exist and represent a limiting factor for a broad use of these strategies (8, 9). Moreover, it is worth considering that the use of integrating vectors comes with significant safety issues. Recently, the occurrence of malignant transformations has been reported in two SCD patients who underwent vector-integrating gene therapy. One of these patients presented leukemic blasts carrying a lentiviral vector insertion site, unveiling the possible pathogenetic role of insertional mutagenesis events (10, 11). Although the actual causal link of these events to the transduction is yet to be proven, these cases caused a partial suspension of clinical trials in Europe and the U.S, highlighting the need of a better comprehension of vector-integrating strategies security profile (12, 13). From this perspective a clear need for safer option emerges and gene editing techniques appears alternative approaches. Programmable nucleases are chimeric molecules composed of two portions: (i) a DNA-binding structure, either RNA or a protein, and (ii) an effector protein domain, capable, through its nuclease activity, to induce a double-strand break (DSB) on the DNA within or in proximity to the binding site. Since their introduction, 4 classes of nucleases have been described: Meganucleases, Zinc Finger Nucleases (ZFNs), Transcription Activator-Like Effector Nucleases (TALENs), and Clustered Regularly Interspaced Short Palindromic Repeats (CRISPR)/Cas9. This last class, thanks to its peculiar DNA-gRNA base-pairing, is the more efficient one. CRISPR/Cas9 system creates DSB at a specific genomic locus followed by recruitment of DNA repair mechanism. Among the others, two are those that are mainly involved in genome editing strategies: (i) non-homologous-end-joining (NHEJ), a homology-independent pathway that involves the alignment of only one to a few bases at most for the re-ligation of two ends; (ii) homology directed repair (HDR) that involves longer stretches of sequence as template to repair DNA lesions.

## 2. Nuclease-based correction of SCD-causing mutation

In the last decade, several gene editing strategies have been investigated in order to revert the SCD-causing mutation. From a historical perspective, in 2014 Genovese and colleagues were the first to attempt to exploit HDR-driven gene repair in human HSCs. Their protocol involved the use of mRNA electroporation and Integrase-Defective LV (IDLV) for delivery of ZFNs and donor DNA template into human cord blood (CB) CD34 + cells (14). Similarly, in 2015 Wang et al. reported homology-driven genome editing in human HSPCs using ZFNs mRNA and Adeno-Associated Virus 6 (AAV6) donor templates (15). In the same year, Hoban et al. demonstrated efficient target cleavage of the β-globin locus, with minimal off-target modifications in HSPCs. Their protocol included the co-delivery of an integrase-defective lentiviral vector or a DNA oligonucleotide as the homologous donor template, and a specific ZNFs programmed to target the pathogenic mutation of SCD (16). In 2016, Dever et al. reported the first CRISPR/Cas9 gene-editing platform for achieving HDR at the *HBB* gene in HSCs derived from mobilized peripheral blood (mPB) by combining Cas9 ribonucleoproteins (RNP) and rAAV6 as homologous donor (HR) delivery (17). The efficiency of this HDR-mediated correction strategies *in vitro* ranged from 7% to 50%, depending on different editing tools and donor delivery systems, and were sufficient to produce clinically-relevant amount of HbA (up to 50% of total Hb), ameliorating the SCD cell phenotype *in vitro* (18). However, after xenograft experiments, the percentage of gene correction detected *in vivo* dropped to less than 10% (19). This evidence showed that HDR is less efficient in quiescent long-term repopulating HSCs, which constitute a minor fraction of the HSPC population. Moreover, all these studies reported that, in parallel with the incomplete HDR-mediated gene repair, DSBs were being repaired through NHEJ, resulting in the formation of small insertions/deletions (InDels) at the *HBB* gene. This occurrence would be particularly detrimental as it can lead to the formation of altered and unstable Hb variants, up to the generation of clones completely unable to produce hemoglobin, functionally similar to the *β*^0^-thalassemia ones (20, 21). Strategies to increase HDR efficiency in HSCs showed encouraging results and hold promise for clinical application of gene correction strategies based on HDR (22, 23). Recent advances for HDR-mediated correction of SCD mutation in human HSPCs have been recently reported by Lattanzi et al. using *ex vivo* Cas9-RNP and rAAV6 based donor DNA. In this study, modifications in culture conditions were introduced. Namely, the addition of UM171 molecule to the culture media and the implementation of the low-density culture conditions in order to promote HSC cycling hence increasing the editing efficiency. Their strategy obtained a high percentage of gene corrected *HBB* (gcHBB) alleles *in vitro* and favored the long-term engraftment (up to ∼4-fold) of human cells with gcHBB alleles *in vivo* (24). Moreover, the percentage of human engraftment and gcHBB alleles were even higher in HSPCs derived from SCD patients compared to healthy donor HSPCs (24). Recently, given the observation that DSB-induced Indels can be predicted, Bodai et al. proposed a “double tap” system in order to improve HDR-based strategies. The latter is based on a primary gRNA—specific for a genomic sequence—and on secondary gRNAs targeting the predicted NHEJ-induced Indel sequences, hence improving the HDR-mediated genome editing efficiency (25). While encouraging, it is still unclear whether the efficiency rates of HDR-mediated *HBB* gene correction could enable effective clinical applications for SCD.

## 3. Genome editing strategies based on fetal hemoglobin reactivation

High expression levels of fetal hemoglobin (*α*_2_γ_2_, HbF) determine important advantages for SCD patients. In fact, HbF reduces the polymerization process in deoxygenated RBC impairing their sickling (26). This is consistent with two historically-defined clinical observations: (i) newborn patients, in which HbF expression is naturally high, do not present a pathological phenotype and are protected from disease complications (27); (ii) patients who co-inherit large deletions, InDels or point mutations within the *HBG* gene cluster resulting in persistently elevated HbF expression (hereditary persistence of fetal hemoglobin HPFH) are relatively asymptomatic (28–30). Recent years witnessed the development of several genome editing strategies based on reproducing the effects of HPFH mutations. CRISPR/Cas9-mediated reproduction of large deletion within the β-globin locus lead to a strong HbF production by causing a distortion of the locus architecture and a direct interaction with the regulatory elements present in the locus control region (LCR) of *HBG* (31, 32). However, the generation of large deletions requires the simultaneous use of two gRNAs, which might decrease the overall efficiency of the strategy (33). Furthermore, genome editing techniques have been exploited to reproduce the effects of point mutations at the *HBG* genes. HPFH point mutations in the γ-globin promoters, indeed, are known to affect the binding of the two main γ-globin transcriptional repressors, B cell CLL/lymphoma 11A (BCL11A) and leukemia/lymphoma related factor (LRF; also known as ZBTB7A or FBI-1), thus leading to elevated γ-globin expression (34). First attempts of targeting the BCL11A binding site (BS) by CRISPR/Cas9 resulted in an efficient reproduction of a naturally occurring 13-bp HPFH deletion (*Δ*13) at the *HBG* promoter, thus leading to a potent HbF expression (35). Importantly, *Δ*13 has been also observed in cells engrafting in immunodeficient mouse models (36), although this small deletion occurs following microhomology-mediated enjoining mechanism (MMEJ) as DNA repair mechanism, which is less/no-efficient in quiescent HSCs. The persistence of high editing rates, led to the expression of HbF in around half of the cells constituting around 20% of total hemoglobin in *ex vivo* differentiated erythrocytes. More recently, an autologous transplantation study performed on non-human primate model, reported a significant reduction in editing frequency in long-term repopulating HSCs which resulted in a limited γ-globin reactivation (1%–5% over the total β-like globin chains) that might not be sufficient to achieve therapeutically relevant HbF levels (37). Similarly, when the binding site of the other main γ-globin repressor (i.e., LRF) is targeted, a potent HbF production is ensured. However, the InDels frequency at *HBG* promoter in cells repopulating the host BM is lower than the one measured *in vitro* (38). The upregulation of fetal hemoglobin could be achieved by targeting other transcription factors involved in its regulation. Among others, it has been recently reported that knocking-down *ATF4* downregulates the expression of *BCL11A* through lowering the expression of *MYB*. *ATF4* could therefore represent a target for reactivating γ-globin expression (39) However, both *ATF4* and *MYB* have been reported to have several other actions beyond the regulation of HbF in non-erythroid cells (40, 41) underlining the need to identify erythroid-specific regulators.

**Figure 2:**
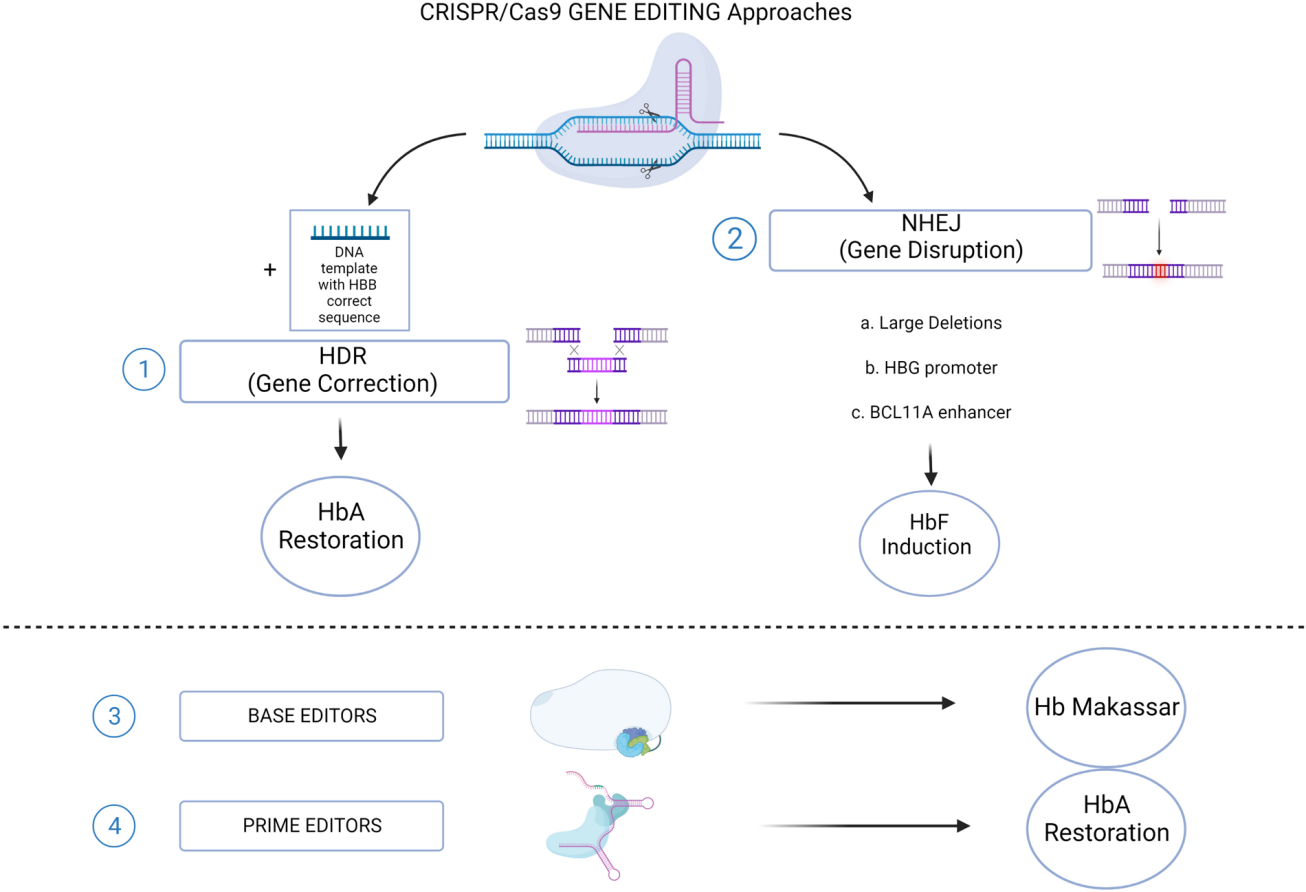
CRISPR/Cas9 genome editing approaches for SCD. (1) The HDR-mediated Gene Correction of the disease-causing mutation, using a DNA Template, leading to HbA restoration. (2) NHEJ-mediated Gene Disruption of target sequences, leading to HbF induction. Different strategies aimed to this goal: a. Large deletions that could either eliminate HbF inhibitory sequences or juxtapose the γ-globin promoters to remote enhancer regions; b. Targeting the *HBG1/2* promoters for the disruption of binding sites for γ-globin transcriptional repressors; c. Disruption of the GATA1 motif in the erythroid-specific intronic enhancer of *BCL11A*, resulting in *BCL11A* knock down. Recently established CRISPR/Cas9-derived editing platforms, i.e. Base editors (3) and Prime editors (4), has been tested to directly change the HbS into a non-sickling variant (Hb Makassar), or to restore the correct HbA protein. (Created with BioRender.com).

Alternative strategies, based on the manipulations of BCL11A expression, have been extensively investigated and represent nowadays the most advanced genome editing-based therapeutic option for SCD patients (Figure 2). To this purpose, the turning point is represented by the identification of erythroid-specific enhancers in BCL11A intron 2 (42). The CRISPR/Cas9-mediated inactivation of an activator binding site in one of the erythroid-specific BCL11A enhancers, induced substantial HbF expression in RBCs without affecting erythropoiesis or BCL11A expression in other lineages (43). Efficacy and safety studies performed in both murine models (44, 45) and non-human primates (46), demonstrated the relevance of this strategy that have been efficiently translated into clinic. The promising results in terms of γ-globin reactivation ensured by CRISPR/Cas9-mediated targeting of BCL11A enhancer and its binding site on *HBG* promoters, gave rise to the hypothesis that a multiplex cutting at the two targets may lead to additional effects on HbF expression when compared to a single one. This hypothesis has been indeed confirmed by the work of two studies that demonstrated that multiplex gene editing led to higher γ-globin expression than single-gene editing without inhibiting erythroid differentiation (47, 48). However it is worth to consider that this kind of approaches are linked to the occurrence of chromosomal translocations between the two cutting sites (49).

## 4. Nuclease-linked safety concerns

As soon as genome editing technology has been introduced, and similarly to insertional mutagenesis associated with integrating vectors, a number of undesired effects provoke by genome editing tools have been considered as major concerns when applied in patients’ cells. Nuclease activity driven by sequence recognition, can also exert itself on off-target sites, i.e., gene sites endowed with fairly tight sequence homology to the desired site. NHEJ-mediated insertion/deletion mutations similar to those occurring at the target site can also occur at these off-target sites, with the potential occurrence of structural aberrations. Hence, several methods to predict or detect off-target activity have been developed. First, it is possible to make *in silico* predictions of putative off-target sites, and then characterize them through deep sequencing techniques. Moreover, it is possible to take advantage of experimental procedures that detect off-target activity *in vitro* and in cell-based systems when available. Commonly used *in vitro* methods include CIRCLE-Seq (50), ONE-Seq (51) and NucleaSeq (52), while GUIDE-Seq (53), DISCOVER-Seq (54) and CAST-Seq (55) are prevalently used cell-based approaches. However, the use of these techniques may be limited by the absence of cell lines harboring the disease-causing mutations. Off-target activity is not the only safety issue related with nuclease-based genome editing procedures. Over the year a number of studies reported that deleterious events are associated with DSB formation even if occurring at the only On-target site (56). Among them, large deletions and inversions (55, 57, 58), chromosomal truncations (59), chromothripsis (60), aneuploidy (61), loss of heterozygosity/imprinting (62) have been described in both cell lines and primary cells.

So far, we have described several genome editing strategies for SCD, nonetheless some of them must face with concerns related with the high sequence similarities within the β-globin locus (*HBG1* vs. *HBG2* or *HBB* vs. *HBD*). In fact, the high homology between two paralogous genes harbor the possibility of developing recombinant events mediated by sequence homology between them as well as the off-target frequency (63). To demonstrate this, such rearrangements were confirmed in *HBB*-edited cell (16, 55, 64). Furthermore, the simultaneous targeting of *HBG1* and *HBG2* was reported to result in deletion of the 4.9 kb region between the two target sites, eliminating *HBG2* in 5%–30% of cells (36, 38). In addition to this, the possibility of complete loss of the short arm of chromosome 11 has been demonstrated in HSCs following the use of CRISPR-Cas nucleases targeting either *HBB*, *HBD* or *HBG1/HBG2* (62). These complications unveiled important safety issues on the use of nuclease-mediated genome editing strategies and led to the development of editing platforms free from DSBs formation.

## 5. DSB-free technology

### 5.1 Base editing

Base Editing is a new gene editing technology that uses a modified CRISPR-Cas9 platform to induce point mutations on the target DNA sequence without inducing DSBs. Two are the main classes of BE enzymes developed cytidine base editors (CBEs), allowing C > T conversions (65, 66) and adenine base editors (ABEs), allowing A > G conversions (67). Given the excellent promise of these tools, they have been further developed in order to broaden their efficiency and specificity, extend their accessibility to impervious gene loci, perform multiplexing, and in parallel trying to maintain a reduced rate of Indels (68, 69).

For SCD, the base editing technology is currently tested for both the correction of the SCD mutation and the induction of fetal hemoglobin. Newby et al. developed an adenine base editor (ABE8e-NRCH) to convert the SCD allele (HBB^S^) into the non-pathogenic variant Makassar β-globin (HBB^G^). *Ex vivo* delivery of mRNA encoding base editor with a targeting guide RNA into HSPCs from SCD patients resulted in 80% HBB^S^-to-HBB^G^ conversion. High editing frequency (68%) have been reported after transplantation of edited human HSPCs into immunodeficient mice indicating a stable editing and moreover, human engrafted cells demonstrated important phenotypical amelioration (decrease hypoxia-induced sickling) thus leading to near-normal hematological parameters and reduced splenic enlargement compared to controls (70). Similarly, Chu et al. designed an ABE-based strategy aim to induce a change in *HBB* point mutation, thus converting HbS to the benign HbG-Makassar in *ex vivo* edited HSCs (71). These results indicate an effective use of base editors in converting the sickling phenotype of SCD into a non-sickling one, with a lasting effect after transplantation and valid bone marrow reconstitution.

Base-editing platforms have also been exploited to reactivate HbF synthesis, either through the generation of HPFH mutations or through the downregulation of BCL11A expression. It is noteworthy that, differently from CRISPR/Cas9, base editing can be used for the generation of mutations creating binding sites (BSs) for transcriptional activators. For example, ABEs have been used to reproduce the −198 T > C HPFH mutation in the *HBG1/2* promoters, creating a BS for the transcriptional activator KLF1 resulting in a 3.5-fold increase in γ-globin expression in HSPC-derived erythrocytes (72). The therapeutic potential of KLF1 BS have been further confirmed by a study of Antoniou and colleagues in which a fine dissection of the contribution of several cis-regulatory elements at the −200 region of the *HBG* promoter have been conducted (73). Alternatively, a potent γ-globin reactivation on healthy donors β-thalassemic HSPCs have also been obtained after reproducing the −115C > T and −114 C > T HPFH mutations, in the *HBG* promoters through ABE (74). Finally, an important phenotypic effect of pathological reversion, with a decrease in the number of sickle RBCs, was achieved by CBE-mediated editing of GATA1 BS in the BCL11A enhancer. The editing percentage of SCD HSPCs was very high in this case and their erythroid progeny exhibited high HbF levels (up to 32%) (75). As mentioned earlier, the use of base editors, with their DSB-free mechanism, allows the possibility of multiplexing, without the risk of unwanted genetic rearrangements. Zeng et al. exploited this potential by simultaneously correcting the β-thalassemia-causing HBB −28 (A > G) mutation and disrupting the GATA1 BS in the BCL11A enhancer. They thus demonstrated, not only that this approach is feasible, but also that it yields better results than individual strategies (75). The applicability of this strategy is yet to be proven in SCD though. Altogether these strategies seem promising in terms of efficacy, even if they need confirmation in terms of safety and applicability before being translated into a clinical scenario.

### 5.2 Prime editing

Riding the wave of success in developing DSB-free editing platforms, Anzalone and colleagues developed a cutting-edge technology with the goal of overcoming the limitations of base editors. Specifically, they developed a tool that allows installation of all types of targeted DNA base pair substitutions, small insertions, small deletions, and combinations thereof, without the need to deliver a donor DNA template (76). These tools have been called “prime editors” (PEs) and structurally consist of a Cas9 nickase coupled to an engineered reverse transcriptase, plus a guide sequence, called pegRNA (prime editing gRNA), that dictates both the start site and the correct sequence to achieve all kinds of desired modifications. With this tool, base conversions, or insertions/deletions of up to 80 bp have become feasible. Also the disease-causing A > T transverse mutation of SCD has been targeted with PEs in HEK293T cells (76), providing a proof of principle on the feasibility of this procedure, previously impossible with currently available Bes. However, despite its great potential, the effectiveness of prime editing has been questioned several times. Chen et al. reported that DNA mismatch repair (MMR) impedes prime editing and promotes undesired InDels byproducts (77). Thus, the same group developed modified prime editing systems in which transient expression of an engineered MMR-inhibiting protein enhanced the editing efficiency and outcome purity while editing the SCD genomic site in human iPSC (77). Following studies have been focused on the optimization of pegRNA by introducing a 3′ structured motif protect the reverse transcriptase template (RTT) from exonuclease degradation, resulting in 3–4 fold PE increased efficacy in several cell lines (78). In the wake of these improvements, Everette and colleagues recently reported the correction of the SCD allele (HBB^S^) to wild type (HBB^A^) at frequencies of 15%–41% in HSPC from patients with SCD (79). Importantly these editing results have been confirmed in human cells retrieved 17 weeks after transplantation into immunodeficient, reporting minimal off-target activity. As result, HSPC-derived erythrocytes carried less sickle hemoglobin, contained HBB^A^-derived adult hemoglobin at 28%–43% of normal levels and resisted hypoxia-induced sickling (79).

## 6. *In vivo* genome editing

An even more recent field of research explored the possibility of editing HSPCs through the *in vivo* delivery of the editing machinery. This strategy could virtually allow easier access to editing procedures since the absence of *ex vivo* manipulation of HSPCs would guarantee lower cost and shorter time frames (Figure 1). Moreover, it would guarantee minor risks in terms of safety as it would not require bone marrow conditioning regimens. Attempts have been made to develop *in vivo* HSC transduction/selection technology using non-integrating adenovirus. *In vivo HBG*-promoter editing by CRISPR/Cas9 in β-YAC/CD46-transgenic mice has been performed (80). The *in vivo* transduction of HSPCs necessarily requires their mobilization towards the peripheral blood, with subsequent intravenous injections of the adenovirus vector, since the direct transduction of bone marrow HSC has proved to be inefficient. In this way it has been possible to obtain the reactivation of human γ-globin in erythrocytes of adult animals, and this result was maintained even after secondary transplantation of HSPCs. Furthermore, once transduced in the peripheral blood, HSCs are able to relocate in the bone marrow, maintaining their self-renewal capacity and thus ensuring a long-term effect (80). Recently, the same group achieved the correction of almost 40% of HBS alleles in HSCs using prime-editor-expressing helper-dependent adenovirus in a SCD mouse model (81). While promising, *in vivo* gene editing for curing SCD poses many technological limitations that need to be addressed. First, it is necessary to strike the right balance between high *in vivo* delivery efficiency and minimal off-target editing of cells and tissues. The employment of viral vectors might represent an efficient strategy for the delivery of molecular gene editing equipment. However, this approach could increase the risk of genotoxicity or immune-response due to the uncontrolled expression of the enzyme or gRNA. On the other hand, the use of non-viral *in vivo* delivery strategies may result in a reduced efficiency with the need of repeated injections (82, 83). Moreover, it is necessary to compare systemic delivery and local injection to determine the best delivery strategy in terms of percentage of edited cells.

## 7. Clinical trials

Gene-editing based therapeutical approaches for SCD have achieved promising results in pre-clinical studies. Hence, curative strategies for SCD patients based on *ex vivo* gene-editing of autologous HSCs have been evaluated into numerous clinical trials in the last years. Most of the first trials are based on the knock-out of the regulatory sequence of the erythroid enhancer of the BCL11A. This approach aims at re-activating the fetal hemoglobin, thus provoking an amelioration of the clinical phenotype in SCD, but also in other β-hemoglobinopathies. Two trials (NCT03745287 and NCT03655678) by Vertex Pharmaceuticals Inc. and CRISPR Therapeutics are evaluating the same CRISPR/Cas9-based product (CTX001) for SCD and TDT β-thalassemia. The first promising results of this product revealed (84) high levels of allelic editing in bone marrow (up to 80% edited cells) and peripheral blood (around 60% edited cells). The first SCD treated patient showed high levels of both total Hb (around 14 g/dl), and HbF fraction, achieving transfusion independence and elimination of Vaso-occlusive episodes (VOCs) for more than one year after the treatment. Recently, Locatelli et al. confirmed these hopeful results by reporting sound data on 31 SCD patients treated with CTX001. All the treated subjects achieved stable and elevated HbF levels, with a complete resolution of VOCs (85). Other two trials have been currently exploring a similar CRISPR/Cas9-mediated HbF reactivation approach, one by Bioverativ (NCT03653247) and the second one by Novartis Pharmaceuticals in collaboration with Intellia Therapeutics (NCT04443907). For this last trial, promising data in terms of sustained induction of fetal hemoglobin and clinical in three patients with SCD have been recently reported (86).

Furthermore, there are two clinical trials evaluating HDR-based gene-editing curative approaches for SCD, aiming at directly correct the pathogenetic mutation in *HBB* gene. The phase 1/2 CEDAR clinical trial (NCT04819841) by Graphite Bio proposed a strategy based on the correction of the SCD mutation using CRISPR/Cas9 and rAAV6 as HDR template (GPH101), (17, 24). Similarly, Walters and colleagues started a phase 1/2 clinical trial evaluating the efficacy of a CRISPR/Cas9 editing system in which the HDR template is delivered by a single-stranded oligodeoxynucleotides (ssODNs) (NCT04774536). However, Graphite Bio recently paused the CEDAR trial because of the occurrence of severe pancytopenia in the first enrolled patient who received GPH101 (https://ir.graphitebio.com/press-releases/detail/84/graphite-bio-announces-voluntary-pause-of-phase-12-cedar). Hence, a long-term follow-up of the treated patients is necessary in order to detect possible long-term consequences due to genotoxic events. Lastly, in 2022 Beam therapeutics started a phase 1/2 clinical trial evaluating the efficacy of a base-editing strategy able to increase HbF production in HSCs of SCD treated patients. In details, an ABE induces a base swap in the HBG1/2 promoters, thus re-activating gamma-globulin production without the occurrence of CRISPR/Cas9-induced DSB. So far, this trial represents the first clinical application of a nuclease-free gene editing strategy for SCD.

## 8. Conclusions

As discussed, the field of genome-editing founded in SCD a unique and fertile soil for the development of its clinical potential. Beside from the monogenic nature of the disease, the reasons of this success rely on (i) the relative frequency of the patient affected worldwide, (ii) a deep knowledge of the globin gene regulation (iii) the presence of a conspicuous history of previous classical gene therapy approaches. On the other hand, the studies on genome editing provide new insights at both biological and clinical level, creating a thriving loop for bench-to bedside information. Differently from classical gene therapy approaches, where a continuous expression of the therapeutic gene is required, genome editing-based strategies retain the potential to ensure clinical benefits after a single delivery of genome editing tools. Moreover, such strategies profit of the endogenous gene regulation machinery, thus conferring additional safety and efficacy advantages. The promise of transplanting autologous and gene-corrected HSC raised great interest in many research groups worldwide. Preclinical results have demonstrated the feasibility of genetically correcting SCD HSPCs by *ex vivo* as well as *in vivo* strategies (16, 24, 81). The limited contribution of HDR in repairing DSBs generated by the different nuclease-mediated tools in long-term repopulating HSC initially hampered the definitive clinical translation of this strategy for the treatment of SCD. Nevertheless, during the last years these approaches have been refined and improved, accomplishing significant results in further pre-clinical evaluation studies (24). Hence, clinical trials based on HDR-based gene-editing for SCD recently started (NCT04819841 and NCT04774536). Upcoming results will elucidate on the real effectiveness of these curative approaches. Undoubtedly, the gene-editing strategies for SCD based on HbF reactivation are currently the more advanced approaches in terms of clinical translation, achieving very promising results in ongoing clinical trials (NCT03745287 and NCT03655678) (84). Although some studies have hypothesized potentially dangerous effects of high levels of HbF, such as increased risk of cerebral vasculopathy (87), no detrimental effects due to high levels of HbF have been reported so far in subjects with HPFH (88) or in patients treated with strategies aimed at knock-down of BCL11A. However, a factual risk of these strategies (both HDR- and NHEJ-mediated) is linked with the formation of DSBs. Off-target Cas 9 nuclease activity can result in disruption/alteration of normal gene function, with often unpredictable consequences. Even worse, larger-scale chromosomal rearrangements and genomic instability can occur, and their outcome are hardly predictable. These serious concerns in human gene therapies still persist, though the utilization of high-fidelity Cas9 which reduces but is capable of abolishing off-target editing (89). Therefore, better systems for detecting and quantifying these aberrant events are needed. These concerns seem to be particularly relevant for diseases like SCD for which the hematopoietic compartment is also affected by a chronic inflammation status (90). The recent introduction of DSB-free technology paved the way in overcoming such limitations and, especially for prime editing technique, it gave a strong pulse into direct research activity towards the transplantation of gene corrected HSPCs over γ-globin reactivated ones. Recently, these new base-editing systems demonstrated their efficacy in cell lines as well in HSPC (79), unveiling the possibility to translate DSB-free approaches in clinical setting, with several advantages over the other corrective strategies. In fact, reverting the SCD allele back to wild-type (WT) represents the most straightforward approach; relying on the direct elimination of HBB^S^ allele, in contrast with other strategies such as the HbF re-activation. These points, together with the limited risk of InDel occurrence and the minimal off-target activity, outline the nuclease-free correction strategies aimed at directly correct the HbS pathogenic mutation as the most promising genome editing approaches for tackling SCD.
